# Determining the presence of Peripheral Arterial Disease in patients with Rheumatoid Arthritis

**DOI:** 10.31138/mjr.28.2.86

**Published:** 2017-06-27

**Authors:** Andrea C. Grech, Alfred Gatt, Andrew A. Borg, Cynthia Formosa

**Affiliations:** 1 Department of Health, Malta,; 2 Faculty of Health Sciences,; 3 Faculty of Medicine and Surgery, University of Malta

**Keywords:** Rheumatoid arthritis, ankle brachial pressure index, cardiovascular disease risk factors, screening

## Abstract

**Objectives:**

The aim of the study was to determine the manifestations of PAD in a population of RA participants with no history of cardiovascular events.

**Methods:**

A prospective observational non-experimental study was conducted on 100 participants presenting with RA and no history of significant cardiovascular events. Vascular assessment including Doppler spectral waveform analysis and Ankle Brachial Pressure Index was conducted.

**Results:**

Triphasic waveforms was found in the Posterior Tibial Artery (PT) in 70% right foot, 66% left foot and Dorsalis Pedis Artery (DP) in both feet in the64% of the patients. Twenty-nine per cent of the participants had biphasic PT right foot and 33% had biphasic PT left foot. Thirty-six per cent had biphasic DP both feet whilst only one participant (1%) had a discontinuous monophasic PT of both feet. The ABPI readings were found to be normal in 96% of participants and mild PAD was found in only 4% of the study population.

**Conclusions:**

Results indicate that whilst the ABPI index was normal in the majority of participants, waveform analysis was suboptimal (biphasic) in approximately one-third of the study sample. These findings highlight that the assessment of peripheral arterial perfusion should utilize both modalities to identify patients with early PAD.

## INTRODUCTION

Rheumatoid arthritis (RA) is a complex inflammatory disease characterized mainly by systemic inflammation, persistent synovitis and auto antibodies. ^1^ Rheumatoid arthritis has been described as one of the most severe and common conditions across the spectrum of inflammatory rheumatic conditions ^2^ with cardiovascular disease (CVD) representing an extra-articular manifestation associated with increased morbidity and mortality. ^3^

Atherosclerosis is common in RA ^4^ and is associated with the chronic disease-related inflammation involving activation of T-lymphocytes and macrophages and the production of pro-inflammatory cytokines. ^5^ As a result, RA patients exhibit a higher risk of peripheral arterial disease (PAD) compared to the general population. ^6^ Although PAD has a pivotal role in the development of vascular disease especially in the field of diabetes mellitus, ^7–9^ in RA, this entity appears to be under-diagnosed. ^10,11^

Although screening for PAD is not routinely performed in RA subjects, diagnostic measures such as Ankle Brachial Pressure Index (ABPI) and Doppler Spectral Waveform Analysis ^12^ are widely used to investigate such abnormalities in diabetic and other high-risk populations. Of note, ABPI measurement has been reported as an excellent marker to predict cardiovascular disease and mortality. ^13,14^

Since atherosclerosis may remain clinically silent for many years, ^15^ early identification of subclinical PAD is of utmost importance in RA population. Therefore, the aim of the study was to determine the manifestations of PAD in a well-characterized population of RA individuals with no history of cardiovascular events.

## SUBJECT SELECTION AND METHODS

### Subject Selection

Rheumatoid arthritis individuals were recruited from out-patient Rheumatology Clinic in our centre. A hundred consecutive subjects were enrolled for this investigation, on a “first through the door basis”. The study protocol was approved by the University Research Ethics Committee and all participants provided informed consent before data collection. All investigations were carried out in accordance with the principles of the Declaration of Helsinki as revised in 2000. Participants eligible for this study were adults aged >18 years, diagnosed with RA according to the 2010 Revised American College of Rheumatology and European League Against Rheumatism (ACR/EULAR) Diagnostic criteria ^16^ and with no known history of CVD events. Patients with history of diabetes mellitus, ulcerations or amputations or revascularization surgery as well as those on treatment with anti-platelet and anticoagulant regimens were excluded from the study.

### Study Design

A prospective non-experimental observational study was conducted. The clinical tools used during this research were based on validated and previously published methods ^17^ following a thorough review of the literature on international guidelines and recommendations. A database was constructed to record all the information.

### Methods

#### Patient Characteristics

After informed consent, participants’ characteristics were recorded, including gender, age, duration of RA and Body Mass Index. In addition, medication history, blood tests including C-reactive protein, erythrocyte sedimentation rate, rheumatoid factor, anti-cyclic citrullinated peptide antibodies and lipid profile were documented. Hypertension and hypertensive therapy together with additional information such as smoking were also recorded.

#### Peripheral arterial disease

Vascular assessment including Doppler spectral waveform analysis and ABPIs were conducted. The testing modalities and examination methods were carried out by the same experienced investigator (AS), who had over 10 years’ experience in the field, to ensure uniformity. Room temperature where assessment took place was kept at 21 to 23 degrees Celsius (68 to 75 F) to avoid vasoconstriction or vasodilatation of digital arteries due to ambient temperature.

All participants underwent both measures because, although ABPI is the accepted ‘gold standard test’ for diagnosing PAD, we have clearly demonstrated that it is not the case especially if the patients have calcification of arteries. In fact spectral waveform analysis may detect patients with PAD when ABPI may fail to do so. ^18^

Participants were asked to undo all tight clothing around the waist and the arm. Measurements were carried out after a 5-minute rest in a supine position with the upper body as flat as possible. The following procedure was utilized to measure both brachial systolic pressure and dorsalispedis and posterialtibial pressures and to acquire the qualitative Spectral Waveforms.

The Huntleigh® DopplexAssist vascular package (Cardiff, UK), which is composed of a continuous wave Doppler with an 8MHz probe, was used to measure the resting ABPI and to acquire the qualitative Spectral Waveforms of the posterior tibial and dorsalis pedis arteries. The probe was held steadily on the anatomical artery location at an angle between 45 to 60 degrees against the flow of arterial blood until an optimum Doppler signal was achieved. Interpretation of arterial spectral waveforms results was based on evidenced criteria obtained from the literature. ^19^ Waveforms were classified as triphasic, biphasic, monophasic discontinuous and monophasic continuous. Triphasic waveforms were considered normal, whereas the biphasic, monophasic discontinuous and monophasic continuous waveforms were considered as abnormal and indicative of PAD. ^19^

Values were interpreted according to the criteria proposed by the American Diabetes Association. ^20^ ABPI calculations were interpreted as 0.9–.29 normal, lower-extremity vascular disease was defined as an ankle brachial index < 0.90 in either foot. An ABPI of >1.3 was considered significantly elevated and indicative of vascular calcification. All data were recorded on a spreadsheet designed in Microsoft Excel to group together the information required for interpretation of the results. Statistical analyses were carried out using SPSS Version 22 (IBM, Chicago, Illinois, USA). Normalcy for data was statistically tested using the Kolmogorov-Smirnov test, which indicated that since the p-value exceeded the 0.05 level of significance, data were considered to be normally distributed. Thus, the one-way Analysis of Variance (ANOVA) was used to analyse the data.

## RESULTS

A total of 100 participants, including 16 males and 84 females were recruited in this study. The mean age of the study group (±SD) and duration of RA was 61 ±11.2 and 12.2±10.8 years respectively ( **Table 1** ).

**Table 1.** Metabolic parameters of the study population (N=100).

**Metabolic parameters**						
		Frequency (n=100)	Percent	Valid Percent	Mean Duration Years	SD
Gender	Male	16	16	16		
	Female	84	84	84		
BMI Category	Normal	32	32	32		
	Overweight	34	34	34		
	Obese	34	34	34		
Hypertension	No	57	57	57		
	Yes	43	43	43	9.058	7.6471
Hypertension Controlled by medication	No	10	10	23.3		
	Yes	33	33	76.7		
	Non-Hypertensive	57	57			
Hypercholesterolaemia	No	70	70	70		
	Yes	30	30	30	4.2417	3.83467
Cholesterol Control	Diet	13	13	41.9		
	Diet & Medication	18	18	58.1		
	No Cholesterol	69	69			
Never smoked	No	34	34	34		
	Yes	66	66	66		
Family member with RA	No	63	63	63		
	Yes	37	37	37		
Family member with Hypertension	No	51	51	51		
	Yes	49	49	49		
Family member with Hypercholesterolaemia	No	67	67	67		
	Yes	33	33	33		
Family member with CVD	No	51	51	51		
	Yes	49	49	49		

**Table 1** highlights the metabolic characteristics of the study population.

## WAVEFORM SPECTRAL ANALYSIS

When analyzing the Posterior Tibial (PT) Artery, 70% of the participants had triphasic waveforms in the Right foot and 66% in the Left foot. Triphasic waveforms of the Dorsalis Pedis Arteries of both feet were recorded in 64% of the participants.

Twenty-nine percent of participants had biphasic right and 33% had biphasic left Posterior Tibial Arteries. Thirty-six percent had biphasic Dorsalis Pedis of both feet, whilst only one participant (1%) had a discontinuous monophasic PT of both feet.

## ANKLE BRACHIAL PRESSURE INDEX

The ABPI was found to be normal (0.9–1.29) in 96% of participants, whilst mild obstruction was found in only 4% of the subjects (mean 0.85, range 0.82–0.88).

The One-way ANOVA (Analysis of Variance) was used to analyze the investigated co-variates related to cardiovascular disease against the ABPIs of the Right and Left foot independently. The cardiovascular risk factors analyzed included gender, BMI, hypertension, hypercholesterolemia, smoking and RA medications, amongst others. Only hypercholesterolemia was found to be significantly related to the ABPI (p=0.022) in the Left foot. ( **Tables 2** and **3** ).

**Table 2.** Analysis of the investigated co-variates related to cardiovascular disease against the ABPIs of Left Foot.

		**One-Way ANOVA of ABPI [L]**			
**Predictor**	**Categories**	**N**	**Mean**	**Std. Deviation**	**p-Value**
Gender	Male	16	1.097	0.093	0.157
	Female	84	1.065	0.080	
	Total	100	1.070	0.083	
BMI	Normal	32	1.0512	.07623	0.292
	Overweight	34	1.0752	.07243	
	Obese	34	1.0819	.09654	
	Total	100	1.0698	.08274	
Hypertension	No	57	1.072	0.088	0.757
	Yes	43	1.067	0.076	
	Total	100	1.070	0.083	
Hypercholesterolaemia	No	70	1.082	0.086	0.022^*^
	Yes	30	1.041	0.068	
	Total	100	1.070	0.083	
Never smoked	No	34	1.088	0.089	0.113
	Yes	66	1.060	0.079	
	Total	100	1.070	0.083	
Analgesics	No	96	1.073	0.083	0.075
	Yes	4	0.998	0.041	
	Total	100	1.070	0.083	
NSAIDs	No	92	1.073	0.083	0.201
	Yes	8	1.034	0.077	
	Total	100	1.070	0.083	
DMARDs	No	24	1.058	0.078	0.419
	Yes	76	1.074	0.084	
	Total	100	1.070	0.083	
steroids	No	64	1.069	0.084	0.860
	Yes	36	1.072	0.081	
	Total	100	1.070	0.083	
Biologics	No	75	1.064	0.081	0.204
	Yes	25	1.088	0.086	
	Total	100	1.070	0.083	

**Table 3.** Analysis of the investigated co-variates related to cardiovascular disease against the ABPIs of Right Foot.

**One-Way ANOVA of ABPI [R]**					
**Predictor**	**Categories**	**N**	**Mean**	**Std. Deviation**	**p-Value**
Gender	Male	16	1.097	0.105	0.166
	Female	84	1.064	0.083	
	Total	100	1.069	0.087	
BMI	Normal	32	1.0525	.08337	0.440
	Overweight	34	1.0774	.08869	
	Obese	34	1.0757	.08964	
	Total	100	1.0688	.08720	
Hypertension	No	57	1.067	0.087	0.801
	Yes	43	1.071	0.088	
	Total	100	1.069	0.087	
Hypercholesterolaemia	No	70	1.078	0.094	0.123
	Yes	30	1.048	0.066	
	Total	100	1.069	0.087	
Cholesterol Control	Diet	13	1.046	0.059	0.865
	Diet & Medication	18	1.041	0.080	
	Total	31	1.043	0.071	
Never smoked	No	34	1.075	0.096	0.634
	Yes	66	1.066	0.083	
	Total	100	1.069	0.087	
Analgesics	No	96	1.071	0.086	0.151
	Yes	4	1.007	0.102	
	Total	100	1.069	0.087	
NSAIDs	No	92	1.072	0.085	0.159
	Yes	8	1.027	0.104	
	Total	100	1.069	0.087	
DMARDs	No	24	1.055	0.091	0.362
	Yes	76	1.073	0.086	
	Total	100	1.069	0.087	
Steroids	No	64	1.072	0.089	0.655
	Yes	36	1.064	0.084	
	Total	100	1.069	0.087	
Biologics	No	75	1.060	0.083	0.079
	Yes	25	1.095	0.096	
	Total	100	1.069	0.087	

Further statistical analysis involved the Analysis of Covariance (ANCOVA) linear regression analysis which determined whether any significant differences between ABPI and the risk factors exist ( **Tables 4** and **5** ). The last iteration of the ANCOVA test confirmed hypercholesterolemia as a significant predictor (p=.022), thus confirming the results of the ANOVA. ANCOVA tests for the right foot were statistically insignificant.

**Table 4.** ANCOVA test of Left ABPI against all variables.

**Tests of Between-Subjects Effects (ABPI Left)**					
**Source**	**Sum of Squares**	**df**	**Mean Square**	**F**	**P-value**
Corrected Model	0.134	19	0.007	1.036	0.432
Intercept	1.199	1	1.199	176.351	0.000
Gender	0.008	1	0.008	1.247	0.268
BMI Scale	0.011	2	0.006	0.843	0.434
Hypertension	0.005	1	0.005	0.808	0.371
Cholesterol	0.047	1	0.047	6.914	0.010
NSAIDs	0.001	1	0.001	0.202	0.655
Analgesics	0.012	1	0.012	1.751	0.190
DMARDs	0.007	1	0.007	0.983	0.324
Steroids	0.000	1	0.000	0.063	0.802
Biologics	0.008	1	0.008	1.224	0.272
Never smoked	0.009	1	0.009	1.330	0.252
Age	0.001	1	0.001	0.095	0.759
RA Duration	0.007	1	0.007	0.996	0.321
Total Blood cholesterol	0.001	1	0.001	0.094	0.760
High density Lipoproteins	0.000	1	0.000	0.001	0.975
Low density Lipoproteins	0.000	1	0.000	0.047	0.828
Rheumatoid Factor	0.006	1	0.006	0.934	0.337
C-Reactive Protein	0.009	1	0.009	1.295	0.258
Erythrocyte Sedimentation Rate	0.004	1	0.004	0.635	0.428
Error	0.544	80	0.007		
Total	115.130	100			
Corrected Total	0.678	99			

**Table 5.** The last iteration of ANCOVA test for Left ABPI showing cholesterol as a significant predicator.

**Tests of Between-Subjects Effects (ABPI Left)**					
**Source**	**Sum of Squares**	**df**	**Mean Square**	**F**	**P-value**
Corrected Model	0.035	1	0.035	5.416	0.022
Intercept	94.667	1	94.667	14443.713	0.000
Cholesterol	0.035	1	0.035	5.416	0.022^*^
Error	0.642	98	0.007		
Total	115.130	100			
Corrected Total	0.678	99			

## DISCUSSION

This study evaluated the incidence PAD using ankle-brachial index and printed arterial spectral waveforms in subjects living with RA.

Our findings highlight the limitations of the use of ABPI measurements in isolation to detect PAD in patients with RA. Results from the current study demonstrate inconsistencies when utilizing both ABPI and arterial spectral Doppler waveform analysis, as Doppler waveforms were different from ABPI interpretations in most of the recruited subjects. Despite a ‘normal ABPI’ examination in most of the patients, waveform analysis showed impaired vascular function as one third of subjects demonstrated biphasic waveforms in one or both feet. Results of the present study have demonstrated that only 1% of the recruited subject population had severe PAD, which could possibly be symptomatic. On the other hand, 29% of patients presented with biphasic waveforms indicating initial stages of subclinical PAD. These observations suggest that almost one third of RA patients may suffer from early PAD and the silent clinical presentation may lead to underdiagnosis of this condition in this population.

An important characteristic of the recruited sample is the lack of significant co-morbidities, which is the crucial factor when analysing the results of this study. We took care to exclude patients with co-morbidities such as diabetes, treatment with anti-platelet and anti-coagulant treatments, a history of ulcerations or major cardiovascular events in order our sample to include individuals with low CVD risk, thus leaving RA as the main independent variable. The literature identifies CVD risk factors that are common to both diabetes and RA. ^21^ It has also been reported that RA patients have a higher risk of PAD than the normal population. ^6^ Normally, production of new blood vessels may occur either by endothelial sprouting from pre-existing angioblasts (Angiogenesis) or by peripheral recruitment of the endothelial progenitor cells (EPCs) (vasculogenesis). Such procedures are important to maintain healthy tissue and also as compensatory methods for development of collateral circulation in regional ischaemia. The quantity of EPCs in the peripheral blood is reported to be inversely correlated with cardiovascular risk. ^22^ In active RA there is a decrease in circulating EPCs, resulting in increased susceptibility to vascular dysfunction. This is due to the fact that, if less EPCs are found, less vasculogenesis may occur leading to less blood vessel formation and thus increased CVD risk. Wolfe & Michaud confirmed ^23^ that, in RA, functional and numerical EPC decline is attributed to the upregulated production of tumor necrosis factor-alpha as well as other mediators of inflammation that are known to be pathogenic in RA. This is further augmented by high grade systemic inflammation which accelerates vascular risk in RA ^24^ and partially explains the peripheral vascular dysfunction noticed in our highly selective RA population. Since TNF is increased in RA patients’ suppression of systemic inflammatory load with biologic targeted therapies may lead to normalization of EPC and reduction of CVD risk. It could be speculated that the beneficial effects of biologic therapies on CVD risk described over the last years ^25^ may be - amongst others - associated with the attenuation of systemic inflammations’ adverse action on EPC numbers and functionality.

This study has shown that in a cohort of patients with RA with very minimal co-morbidities, nearly all participants (96%) presented with normal ABPI readings. However, qualitative Spectral Waveform Analysis demonstrated suboptimal/mild arterial perfusion in one third of the study group indicative of PAD.

These findings are congruent with those of Chuang et al. ^6^ who reported that RA patients with various co-morbidities showed a significantly higher risk of PAD when compared with their controls without co-morbidities. In contrast, in a case-control study, the authors reported a higher prevalence of abnormal ABPI in patients with RA when compared to matched healthy individuals (p=0.001). ^10^ These inconclusive results provided the rationale for the conduction of our study to investigate whether RA is an independent risk factor for peripheral arterial disease. ^11^ This study now suggests that RA may be an independent risk factor for PAD. It has been reported that Doppler analysis offers a particular advantage over the ABPI, since the detection of a pulsatile flow using Doppler analysis may be possible even in calcified arteries. Doppler waveform analysis therefore allows the detection of early arterial disease when normal ABPI readings are recorded. Our observations also indicate that further physiological testing such as toe pressure and toe brachial pressure indices should be performed to establish whether PAD is actually present in case those inconsistencies occur between two screening modalities for PAD namely ABPI and Doppler analysis. Earlier diagnosis of PAD in RA allows the prompt management of CVD risk factors and CVD risk stratification which improves long term outcomes in this population. ^26^

The duration of RA does not seem to have any impact on arterial perfusion since in this present study, although the duration of the condition amongst our participants ranged from 2 to 24 years, no significant differences was found between RA duration and ABPI and Doppler Spectral Waveform analysis. The only statistically significant association established between cardiovascular disease co-variates assessed was between high serum cholesterol levels and ABPI readings (p=0.022).

A limitation of this study is that subjects use of anti-depressants were not recorded, thus authors are unaware of the extent of use of this type of medication, which may affect vasodilation and thus the assessment of vascular supply. More research is required in this field in order to explore the relationship between RA and individual co-morbidities in order to identify which of these factors contribute to PAD in this population.

## CONCLUSION

Results indicated that whilst the ABPI index was normal in the majority of participants, waveform analysis was abnormal (biphasic) in approximately one-third of the study sample. This led to the conclusion that some of the recruited subjects with normal ABPI index but abnormal waveforms could mistakenly be classified as normal. This research emphasizes that assess peripheral arterial perfusion assessment should utilize both modalities and when they do not correlate (with either one suggesting PAD), these patients should be monitored or further evaluated accordingly. Early diagnosis of PAD allows the prompt commencement CVD risk management which may delay long-term complications, improve outcomes and reduce the financial burden which this condition imposes on both patients and the healthcare system. Current recommendations about physiological testing of peripheral perfusion in RA should consider including spectral waveforms as part of the assessment in this group of patients.
